# Comparative Genomic Analysis of Two Clonally Related Multidrug Resistant *Mycobacterium tuberculosis* by Single Molecule Real Time Sequencing

**DOI:** 10.3389/fcimb.2017.00478

**Published:** 2017-11-15

**Authors:** Kenneth Siu-Sing Leung, Gilman Kit-Hang Siu, Kingsley King-Gee Tam, Sabrina Wai-Chi To, Rahim Rajwani, Pak-Leung Ho, Samson Sai-Yin Wong, Wei W. Zhao, Oliver Chiu-Kit Ma, Wing-Cheong Yam

**Affiliations:** ^1^Department of Microbiology, Queen Mary Hospital, The University of Hong Kong, Hong Kong, Hong Kong; ^2^Department of Health Technology and Informatics, The Hong Kong Polytechnic University, Hong Kong, Hong Kong; ^3^KingMed Diagnostics, Science Park, Hong Kong, Hong Kong

**Keywords:** multidrug resistance, *Mycobacterium tuberculosis*, PacBio sequencing, growth rate, comparative genomic analysis

## Abstract

**Background:** Multidrug-resistant tuberculosis (MDR-TB) is posing a major threat to global TB control. In this study, we focused on two consecutive MDR-TB isolated from the same patient before and after the initiation of anti-TB treatment. To better understand the genomic characteristics of MDR-TB, Single Molecule Real-Time (SMRT) Sequencing and comparative genomic analyses was performed to identify mutations that contributed to the stepwise development of drug resistance and growth fitness in MDR-TB under *in vivo* challenge of anti-TB drugs.

**Result:** Both pre-treatment and post-treatment strain demonstrated concordant phenotypic and genotypic susceptibility profiles toward rifampicin, pyrazinamide, streptomycin, fluoroquinolones, aminoglycosides, cycloserine, ethionamide, and para-aminosalicylic acid. However, although both strains carried identical missense mutations at *rpoB* S531L, *inhA* C-15T, and *embB* M306V, MYCOTB Sensititre assay showed that the post-treatment strain had 16-, 8-, and 4-fold elevation in the minimum inhibitory concentrations (MICs) toward rifabutin, isoniazid, and ethambutol respectively. The results have indicated the presence of additional resistant-related mutations governing the stepwise development of MDR-TB. Further comparative genomic analyses have identified three additional polymorphisms between the clinical isolates. These include a single nucleotide deletion at nucleotide position 360 of *rv0888* in pre-treatment strain, and a missense mutation at *rv3303c* (*lpdA)* V44I and a 6-bp inframe deletion at codon 67–68 in *rv2071c* (*cobM)* in the post-treatment strain. Multiple sequence alignment showed that these mutations were occurring at highly conserved regions among pathogenic mycobacteria. Using structural-based and sequence-based algorithms, we further predicted that the mutations potentially have deleterious effect on protein function.

**Conclusion:** This is the first study that compared the full genomes of two clonally-related MDR-TB clinical isolates during the course of anti-TB treatment. Our work has demonstrated the robustness of SMRT Sequencing in identifying mutations among MDR-TB clinical isolates. Comparative genome analysis also suggested novel mutations at *rv0888, lpdA*, and *cobM* that might explain the difference in antibiotic resistance and growth pattern between the two MDR-TB strains.

## Introduction

Tuberculosis (TB) caused by *Mycobacterium tuberculosis* (MTB) has been a global public health challenge for decades. It was estimated that there were 10.4 million new cases and 1.8 million TB-related deaths worldwide annually (World Health Organization, 2016). Despite the enormous global efforts in TB control, multidrug resistant TB (MDR-TB) with resistance to at least rifampicin (RIF) and isoniazid (INH) is starting to threaten the treatment regimens currently available. In 2016, World Health Organization estimated a total of 480,000 TB cases caused by MDR-TB. Among these MDR-TB cases, ~9.5% were extensively drug resistant TB (XDR-TB) with additional resistance toward fluoroquinolones and one of the other second-line injectable anti-TB drugs.

Understanding the molecular basis of drug resistance and the transmission of MDR-TB is critical in developing new therapeutic and preventive strategies against the disease. However, while over 95% of phenotypic RIF resistance can be explained by the mutations at 81 bp rifampicin resistance determining region (RRDR) of *rpoB* (McCammon et al., 2005), *katG* S315T missense mutation and mutations at the promoter region of *inhA* can only explain 50–95 and 8–43% of phenotypic INH resistance respectively (Witney et al., 2016). The discordance between phenotypic and genotypic drug resistance profile suggested that genetic variations among MDR-TB strains are still far from fully elucidated. Whole genome sequencing using short read sequencing platform allows identification of additional resistance-related mutations, but there are still limitations to the current method. First of all, the repetitive nature and high GC content of MTB genome often result in amplification bias during library preparation, which in turn causes fragmented genome assembly (Pop and Salzberg, 2008; Treangen and Salzberg, 2011). Secondly, although there are numerous pipelines for accurate Single Nucleotide Polymorphisms (SNPs) identification, bioinformatics tools in determining other genetic variations such as indels and copy number variations are still very much limited (Ratan et al., 2015; Steglich and Nubel, 2017). More importantly, comparative genomic analyses between drug-sensitive TB and MDR-TB strains often result in thousands of potential mutations. With the vast majority of these mutations being lineage-specific SNPs or neutral mutations accumulated during bacterial replication, identification of novel resistant-related mutations from other irrelevant gene candidates would require massive amount of computation analysis and experimental validations (Fan et al., 2014).

Single Molecule Real Time (SMRT) Sequencing is a recently developed sequencing platform that has greatly simplified the *de novo* assembly step of small microbial genomes (Roberts et al., 2013). With an average of 10–20 kbp per read, SMRT sequencing can easily span across highly repetitive DNA sequences (Scott and Ely, 2015; Zhu et al., 2016). Moreover, library preparation for SMRT Sequencing does not require PCR amplification, which means there would be virtually no GC bias during sequencing step (Ferrarini et al., 2013). In other words, SMRT Sequencing is able to reduce the number of gaps in the final assembled genomes while accurately determine the presence of any complex structural variations. The unbiased GC coverage and the extraordinarily long reads in SMRT Sequencing makes the platform particularly suitable for sequencing MTB genomes with unexceptionally high GC content and large numbers of repetitive regions (Ferrarini et al., 2013).

In this study, we focused on two consecutive phenotypically different MDR-TB clinical isolates collected from the same patient during the course of anti-TB regimen. Using SMRT Sequencing platform, genomic libraries prepared from the two strains were sequenced, *de novo* assembled and analyzed. The clonal relatedness between the two MDR-TB clinical isolates allowed an extensive removal of lineage-specific SNPs by comparative genomic analysis. Mutations responsible for the phenotypic differences could thus be revealed.

## Materials and methods

### Bacterial strains

**Figure 1:**
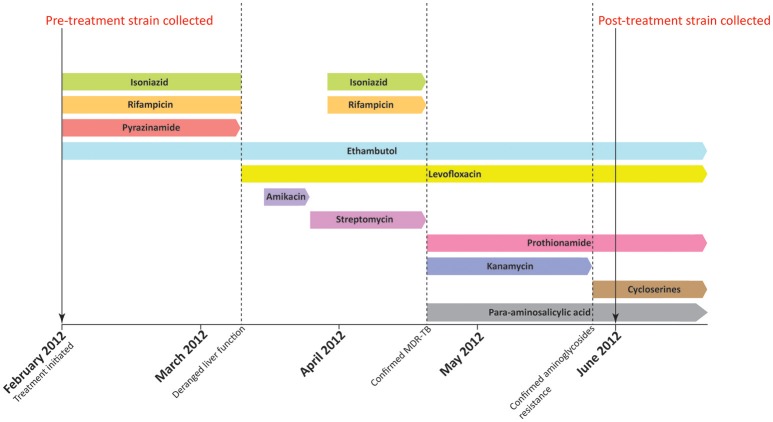
Treatment history of the patient. The patient was initially treated with standard regimen using isoniazid, rifampicin, ethambutol, and pyrazinamide when first diagnosed with MTB infection in February 2012. Pre-treatment strain was collected before the initiation of anti-TB treatment. In early March 2012, the use of isoniazid, rifampicin, and pyrazinamide was halted due to deranged liver function. The regimen was subsequently switched to ethambutol and levofloxacin. Administration of isoniazid, rifampicin was resumed in late March. However, the treatment regimen was changed again in mid-April as the pre-treatment strain was confirmed to be MDR-TB. A combination of ethambutol, levofloxacin, prothionamide, kanamycin, and para-aminosalicylic acid was therefore administered in mid-April. Kanamycin was replaced by cycloserine in late May 2012 as phenotypic drug susceptibility test showed that the pre-treatment strain was resistant to aminoglycosides. The post-treatment strain was subsequently collected in June 2012, which is 4 months after the initiation of anti-TB treatment.

This study has been approved by the Institutional Review Board of the University of Hong Kong/Hospital Authority Hong Kong West Cluster (Ref. number: UW 12–309). The first MTB strain (pre-treatment strain) was isolated from sputum culture of a newly diagnosed TB patient in February 2012. Anti-TB therapy was initiated with standard regimen, but was switched to a combination of levofloxacin and ethambutol due to the deranged liver function in March 2012. (Figure 1) The pre-treatment strain was later confirmed to be MDR-TB with additional resistance toward aminoglycosides by initial phenotypic drug susceptibility tests (DSTs) from sputum culture. The treatment was therefore changed to cycloserine (CYS), levofloxacin, prothionamide, ethambutol (EMB), and para-aminosalicylic acid (PAS). The second MDR-TB strain (post-treatment strain) was collected from the same patient in June 2012, which was 4 months after the initiation of anti-TB therapy. The patient responded to the switched treatment with improved bilateral lung infiltrate in chest radiograph and improved respiratory and laryngeal symptoms. However, the patient passed away in October 2012 due to other complications. All primary MTB cultures were inoculated onto Lowenstein-Jensen medium (BioMérieux SA, France) and bacterial colonies were stored at −80°C prior usage. MTB laboratory strain H37Rv (ATCC 27294) was purchased from American type Tissue Culture Collection (ATCC) as growth controls for phenotypic DSTs, and as reference strain in multi-locus variable number tandem repeat analysis (MLVA). Drug susceptible MTB Beijing/W strain as defined our previous study (Lam et al., 2011) was included as reference strain for growth curve analysis.

### Preparation of MTB culture

Frozen aliquot of MTB strains were inoculated into 10 mL Middlebrook 7H9 medium (BD Bioscience, CA, USA) supplemented with 10% OADC and 0.1% Tween 80. The inoculum was incubated at 37°C in 5% CO_2_. At day-7 post-inoculation, 0.1 mL of the inoculum was spread onto Middlebrook 7H11 agar supplemented with 10% OADC to isolate single colonies.

### Phenotypic DST

Minimum inhibitory concentrations (MICs) for INH, RIF, EMB, Streptomycin (STR), Ofloxacin (OFX), Moxifloxacin (MOX), Kanamycin (KAN), Amikacin (AMI), Rifabutin (RFB), Ethionamide (ETH), CYS, and PAS were analyzed by Trek Sensititre MYCOTB MIC plate (MYCOTB; Trek Diagnostic Systems, Cleveland, OH) according to manufacturer's instruction. Growth in MYCOTB Sensititre plate was monitored at 7, 10, 14, and 21 days post-inoculation. MICs were determined as the lowest antibiotic concentration that causes a significant growth reduction when compared to the positive control well. Pyrazinamide (PZA) resistance was determined by BACTEC™ MGIT™ 960 PZA Kit. Critical concentration of PZA used in MGIT™ 960 PZA Kit was 100 mg/L. Experiments were duplicated to ensure reproducible result for each colony. The tests were also performed on three independent colonies from each strain (pre-treatment strain and post-treatment strain).

### MLVA and spoligotyping

The 24-loci Mycobacterium Interspersed Repetitive Unit-Various Number of Tandem Repeat (MIRU-VNTR) genotyping was performed according to previously published protocols (Weniger et al., 2010) using MTB laboratory strain H37Rv the control strain. Virtual spoligotyping was performed by identifying the presence of 43 unique spacer sequences (Kamerbeek et al., 1997) in the assembled genomes using Blastn (Basic Local Alignment Search Tools).

### *In Vitro* growth rate

Single colony of MTB on Middlebrook 7H11 agar were inoculated into 7H9 broth supplemented with 10% OADC and 0.1% Tween 80. At day 7 post-inoculation, optical density at 600 nm (OD_600_) was checked, and the turbidity of the inoculum was adjusted to OD_600_ = 0.1. A triplicate set of 10 mL bacterial inoculums for each colony were then prepared by adding 500 μL of prepared inoculum into 9.5 mL 7H9 broth supplemented with 10% OADC and 0.1% Tween 80. OD_600_ of the samples were measured every 48–72 h for a total of 14 days. The assay was triplicated using three different colonies from each strain.

Growth curves were generated using GraphPad Prism 6.0 (GraphPad Prism Software, Inc., San Diego, CA). For each bacterial strain, the mean of OD_600_ from three independent biological replicates were plotted against time of incubation. The resultant growths were analyzed using non-linear regression by fitting Gompertz Function into each data set and compared to the growth rate of H37Rv and MTB Beijing/W strain. *P*-values were calculated using the extra sum-of-squares *F*-test.

### Genomic DNA preparation

Genomic DNA was extracted according to previous published protocol (Belisle et al., 2009). In brief, MTB colonies were inoculated into 10 mL 7H9 broth until OD_600_ reached ~1.0. Bacterial pellet was collected by centrifugation and frozen at −80°C overnight. The pellet was resuspended in SET buffer (25% w/v sucrose, 50 mM EDTA and 50 mM Tri-HCl pH 8.0) and digested by lysozyme overnight. Bacterial suspension was then digested by Proteinase K, and genomic DNA was extracted and purified by phenol-chloroform extraction method. Purity of genomic DNA was checked by Qubit Assay. Integrity of genomic DNA was checked by 0.6% agarose gel electrophoresis.

### SMRT sequencing and *de novo* assembly

A total of 15 μg genomic DNA per strain was used for SMRT Sequencing (Pacific Bioscience, USA). A 20 Kbp library was prepared from the genomic DNA, and each library was loaded onto one SMRT cell using MagBead One Cell Per Well protocol. SMRT sequencing was performed using P6-C4 chemistry. Continuous long reads (CLRs) generated by SMRT sequencing were used for *de novo* assembly by Hierarchical Genome Assembly Process (HGAP.2) in SMRT Portal (version 2.3.0), and the resultant assembled contig was further polished with Quiver. Open reading frames (ORFs) were then predicted using Glimmer3, RNAmmer-1.2 and tRNAscan-SE. Functional annotation of ORFs were performed by BLASTall analysis (version 2.2.26) according to the NR database with all species, and only ORFs with a mean similarity of >97% were selected for subsequent analysis.

### Whole genome alignment, variant calling, and resistant-related mutation identification

To access the presence of large-scale evolutionary events in the MDR-TB strains such as genome rearrangement or inversion, the assembled genomes were aligned with MTB H37Rv reference genome (Accession number: AL123456.3) and MTB Beijing reference genome (Accession number: NZ_CP011510.1) by Mauve aligner (version 2.3.1) using the progressiveMauve algorithm. The alignment was performed with matched seed-weight of 15 whereas other mandatory arguments were run on programme default.

To identify the genetic variation between the two MDR-TB strains, sequencing reads of post-treatment strain were aligned with reference to the *de novo* genome of pre-treatment strain for variant calling by Quiver using default parameters. Variants were selected when coverage at the specific site is higher than 40X and Phred quality score was over 45. Identity and the genome coordinate of the confirmed variant were then determined by Blastn using MTB H37Rv strain as reference.

### Sanger sequencing

Sanger sequencing covering resistance-related mutations were performed for both pre-treatment and post-treatment strain as published previously. The sequenced regions included RRDR of *rpoB, katG* codon 315, *inhA* upper promoter region, *embB* codon 306, fluoroquinolones resistant-determining region of *gyrA* and *gyrB* (Starks et al., 2009; Lau et al., 2011; Siu et al., 2011, 2014), and the entire DNA sequence for *ubiA, pncA, rpsL, ethA, rrs, tlyA*, and *folC* (Sreevatsan et al., 1996; Juréen et al., 2008; Jugheli et al., 2009; Machado et al., 2013; Zhao et al., 2014; He et al., 2015). Natural polymorphisms and lineage-specific SNPs were excluded from this analysis.

For regions with differential genetic variations, oligonucleotide primers were designed using Primer3 (version 4.0.0) and NetPrimer for PCR amplification and Sanger sequencing (Table 1). PCR products were generated from genomic DNAs using HotStar Taq *Plus* DNA Polymerase and standard PCR conditions. Consensus sequences were assembled and edited by Staden Package (Version 2.0.0), and were compared to the reference sequences in MTB H37Rv strain by BioEdit (version 7.2.5).

**Table 1:** Oligonucleotide primers designed for confirmatory Sanger Sequencing at novel gene targets with differential mutations.

**Gene**	**Orientation**	**Oligonucleotide sequence**
*rv0888*	Forward	5′-ATGACTTGCTCAATGCCCTG-3′
	Reverse	5′-GGTCACGATGCCATGCTG-3′
*rv2071c*	Forward	5′-GACGGTCTATTTCATCGGAGC-3′
	Reverse	5′-GGCGGTATGGGTGTGGAC-3′
*rv2921c*	Forward	5′-GACGAGGTGTTGCTGGTGC-3′
	Reverse	5′-GGGTCGCCACCATCAGAC-3′
*rv3303c*	Forward	5′-CTAGGTTATGGGCTGTGGTGAC-3′
	Reverse	5′-CTCATGCTCACTGGTGGAG-3′

### Alignment analysis

Amino acid sequences of target gene candidates in MTB H37Rv were identified in KEGG (Kyoto Encyclopedia of Gene and Genomes). Available orthologous sequences of target gene candidates were then searched and downloaded from pathogenic mycobacterial strains in SimilarSequence DataBase (SSDB). The strains were selected in order to include most of the pathogenic mycobacteria species commonly identified in Hong Kong. Multiple protein sequence alignment was conducted using Clustal Omega (http://www.ebi.ac.uk/Tools/msa/clustalo/) and the alignment results were visualized by Jalview (version 2.10.1).

### *In Silico* simulation of mutation effect

The effects of missense mutations on protein stability were predicted using structural-based algorithms. X-ray crystal structures of potential gene candidates were downloaded from RCSB-Protein Data Bank (RCSB-PDB). The protein structure, corresponding amino acid substitution, and chain identifiers were then uploaded onto DUET web server to derive a combined/consensus prediction from mCSM and SDM. The predicted result was expressed in terms of Gibbs Free Energy change (ΔΔG). Amino acid substitutions leading to negative ΔΔG values were regarded as destabilizing mutations and vice versa (Pires et al., 2014). The resultant predicted structure of the mutant protein was downloaded as PDB files, and was visualized by PyMol (version 1.7.2.1).

For indel mutations or target genes without available X-ray crystal structures, effect of mutations were predicted by sequence based algorithms. Protein sequences of target gene candidates were analyzed by Protein variation effect analyzer (PROVEAN) (version 1.1). A score of less than −2.5 was considered as deleterious (Choi and Chan, 2015).

## Results

### Phenotypic DST on both strains

MYCOTB Sensititre assay showed that the MICs for pre-treatment and post-treatment strains were within ±1 doubling dilution in RIF, STR, MOX, OFX, AMI, KAN, ETH, PAS, and CYS which was concordant to the initial phenotypic DST result from sputum culture. MGIT PZA kit showed that both MDR-TB strains were resistant to PZA at 100 mg/L However, the post-treatment strain showed 4-fold elevation in EMB MIC (8–32 mg/L), 8-fold elevation in INH MIC (0.5–4 mg/L), and 16-fold elevation in RFB MIC (1–16 mg/L) when compared to the pre-treatment strain (Table 2).

**Table 2:** Minimum Inhibitory Concentration of 12 anti-TB drugs in pre-treatment and post-treatment strain as determined by MYCOTB Sensititre Assay.

**Antibiotics**	**Minimum inhibitory concentration (mg/L)**	
	**Pre-treatment strain**	**Post-treatment strain**
Rifampicin	>16	>16
Isoniazid	0.5	4
Ethambutol	8	32
Streptomycin	32	32
Ofloxacin	0.5	1
Moxifloxacin	0.25	0.5
Amikacin	4	4
Kanamycin	>40	>40
Ethionamide	20	>40
Cycloserine	16	16
Rifabutin	1	16
Para-aminosalicylic acid	<0.5	<0.5

### MLVA and spoligotyping result

MLVA and *in-silico* spoligotyping were performed to examine the clonal relatedness of the two MDR-TB clinical isolates. Both pre-treatment and post-treatment strains showed identical spoligotyping patterns of Beijing lineage, which is characterized by the absence of spacer 1 to 34. The identical genotyping pattern in 24-loci MIRU-VNTR analysis further proved that the two MDR-TB strains were clonally related (Table 3).

**Table 3:** MIRU-VNTR and Spoligotyping pattern of pre-treatment and Post-treatment strains using MTB H37Rv as reference.

**Strains**	**MIRU-VNTR genotype**																								**Spoligotyping**	**Lineage**
	**MIRU02**	**Mtub04**	**ETRC**	**MIRU 04**	**MIRU 40**	**MIRU 10**	**MIRU 16**	**Mtub 21**	**MIRU 20**	**Qub 11b**	**ETRA**	**Mtub 29**	**Mtub 30**	**ETRB**	**MIRU 23**	**MIRU 24**	**MIRU 26**	**MIRU 27**	**Mtub 34**	**MIRU 31**	**Mtub 39**	**Qub 26**	**QUB4156**	**MIRU 39**		
H37Rv	2	2	4	3′	1	3	2	2	2	5	3	4	2	3	6	1	3	3	3	3	5	5	2	2	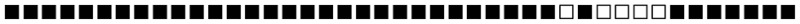	H37Rv
Pre-treatment strain	2	2	4	2	2	3	3	5	2	5	4	4	4	2	5	1	7	3	3	4	5	8	2	3	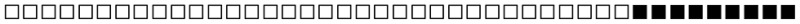	Beijing
Post-treatment strain	2	2	4	2	2	3	3	5	2	5	4	4	4	2	5	1	7	3	3	4	5	8	2	3	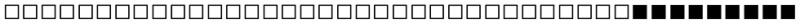	Beijing

### *In Vitro* growth rate determination

**Figure 2:**
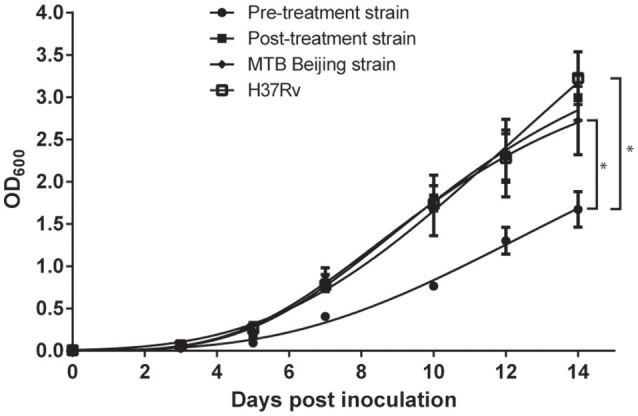
Growth rate of pre-treatment and post-treatment strains measured in a 14-days interval. Bacteria were incubated in 7H9 broth and OD_600_ was measured every 48–72 h in a 14-days interval. *P*-value was calculated by non-linear regression model by fitting Gompertz Function into each data set. Data were the means OD_600_ from triplicated biological replicates ± Standard Error of Mean. ^*^p < 0.001.

The growth fitness of both MDR-TB strains were analyzed by their respective *in vitro* growth rate. H37Rv, MTB Beijing/W strain, pre-treatment and post-treatment strains produced a typical, single-phase growth profiles when incubated at *in vitro* condition. When compared to the H37Rv and MTB Beijing/W strain, the pre-treatment strain showed a significant fitness cost with a 2-fold reduction in bacterial growth (*p* < 0.001). The *in-vitro* growth rate of post-treatment strain was restored and was comparable to that of the MTB Beijing/W strain (*p* = 0.76). The result indicated the potential recovery of growth fitness in the post-treatment strain (Figure 2).

### SMRT sequencing and *de novo* assembly

To identify the genomic characteristics of pre-treatment and post-treatment strain, their respective genomic sequences were analyzed by SMRT Sequencing platform (GenBank Accession Numbers: CP022578 and CP022577). High quality reads (Quality score > 0.8) with a sub-reads N-50 value greater than 11 Kbp were obtained in both analyses. The sequencing depth of pre-treatment and post-treatment strains were 307X and 241X, respectively, with a uniform coverage across the genomes. Subsequent *de novo* assembly generated two fully closed circular genomes without the need for additional gap-filling procedures. While only one single contig was generated in the post-treatment strain, the *de novo* assembly of pre-treatment strains genome resulted in two contigs with a size of 4.41 and 20 Kbp. Further examination at the 20 Kbp contig revealed that the first 10 Kbp sequence was reverse complementary to the remaining 10 Kbp sequence, and it shared 99.9% identity to the 16–27th Kbp region of the 4.41 Mbp contig. In this context, we determined the 20 Kbp contig was a result of extra coverage at the 16–27th Kbp region of MTB genome and was excluded during the final genome assembly. The sizes of both resultant *de novo* assembled genomes were 4.413 Mbp with a GC content of 65.62% (Table 4). A total of three ribosomal RNA, 45 transfer RNA and ~4,000 ORFs were predicted from both genomes. The numbers matched with the MTB H37Rv reference genome.

**Table 4:** Technical details of SMRT Sequencing in pre-treatment and post-treatment strain.

	**Pre-treatment strain**	**Post-treatment strain**
Filtered Subread Bases (bp)	1,373,823,260	1,069,071,955
Subread N50 (bp)	11,565	11,368
Number of Contig	2	1
Polished Contig Total Length (bp)	4,463,392	4,432,733
Coverage	307X	241X
Polished contig length circular (bp)	4,413,669	4,413,712
GC Content (%)	65.62	65.62

### Comparative genomic analysis between the pre-treatment and post-treatment strains

MAUVE alignment was first applied to compare the genomic orders of pre-treatment strain, post-treatment strain, MTB H37Rv strain and MTB Beijing strain. Despite slight differences in the genomic length of Beijing strain reference genome (4.38 Mbp), the four aligned genome showed identical genome order, indicating the absence of gene rearrangement events in both MDR-TB clinical isolates.

The presence of resistant-related mutations were identified from the assembled genomes and confirmed by direct Sanger sequencing. A total of 13 resistant-related genes were examined. Both pre-treatment and post-treatment strains carried identical missense mutations at *rpoB* S531L, *inhA* C-15T, *embB* M306V, *pncA* G162D, *rpsL* R43K, and *rrs* A1401G. No additional resistant-related mutations were found in *katG, ubiA, gyrA, gyrB, tylA, ethA*, and *folC* (Table 5). Comparative genomic analysis further identified a total of 15 polymorphisms between the two MDR-TB clinical isolates. Among these 15 polymorphisms, four were checked by confirmatory Sanger sequencing. A single base-pair frameshift deletion at *rv0888* was validated at codon 120 (nucleotide 360) within the pre-treatment strain. Meanwhile, a valine to isoleucine substitution at codon 44 within Rv3303c (*lpdA*) and a 6 base-pair in-frame deletion at codon 67–68 of Rv2071 (*cobM*) were validated within the post-treatment strain. An 8-bp frameshift deletion at codon 415–418 of *rv2921c* in post-treatment strain was originally identified by SMRT sequencing, but it was proved to be a false variant by Sanger sequencing (Table 5). For the remaining 11 differential polymorphisms identified by SMRT Sequencing, they were indel mutations located within a 100 bp repetitive region of PE-PGRS2 with an exceptionally high GC content (77.6%). Due to the repetitive nature and the high GC content, we were unable to amplify the target region for further confirmatory Sanger Sequencing.

**Table 5:** Comparison of variants identified by SMRT Sequencing and Sanger sequencing in pre-treatment and post-treatment strain.

**Genes**	**Pre-treatment strain**		**Post-treatment strain**	
	**SMRT Sequencing**	**Sanger sequencing**	**SMRT Sequencing**	**Sanger sequencing**
*rpoB*	S531L (tCg → tTg)	S531L (tCg → tTg)	S531L (tCg → tTg)	S531L (tCg → tTg)
*katG*	WT	WT	WT	WT
*inhA*	C-15T	C-15T	C-15T	C-15T
*embB*	M306V (Atg → Gtg)	M306V (Atg → Gtg)	M306V (Atg → Gtg)	M306V (Atg → Gtg)
*ubiA*	WT	WT	WT	WT
*pncA*	G162D (gGt → gAt)	G162D (gGt → gAt)	G162D (gGt → gAt)	G162D (gGt → gAt)
*rpsL*	R43K (aAg → aGg)	R43K (aAg → aGg)	R43K (aAg → aGg)	R43K (aAg → aGg)
*gyrA*	WT	WT	WT	WT
*gyrB*	WT	WT	WT	WT
*rrs*	A1401G	A1401G	A1401G	A1401G
*tlyA*	WT	WT	WT	WT
*ethA*	WT	WT	WT	WT
*folC*	WT	WT	WT	WT
*rv0888*	360delG	360delG	WT	WT
*rv2071c*	WT	WT	199_204delGCCGAC	199_204delGCCGAC
*rv2921c*	WT	WT	1244_1251delATTCGTCG	WT
*rv3303c*	WT	WT	V44I (Gta → Ata)	V44I (Gta → Ata)

### Mutation effect predictions

A single base-pair deletion in *rv0888* was identified in pre-treatment strain. The frameshift mutation would disrupt reading frame and induce a stop codon at codon 137, which truncated the protein from 490 residues to 136 residues. Multiple sequence alignment result showed that the remaining residues in the truncated Rv0888 were only conserved among *Mycobacterium tuberculosis complex* (MTBC) (Supplementary Figure 1)—, whereas the rest of the conserved regions would be removed after the truncation. With more than two-third of the amino acid sequence being removed, the resultant translated product would no longer be functional when compared to the wild-type protein.

**Figure 3:**
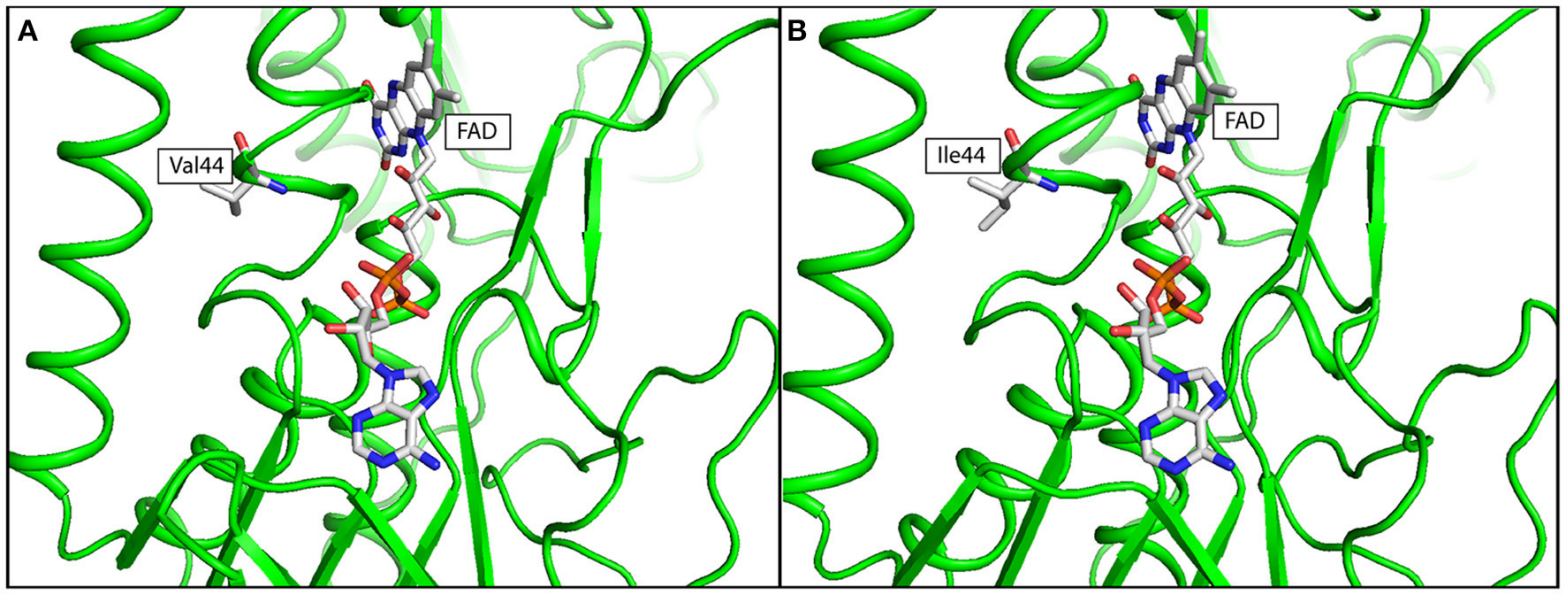
*In silico* simulation of FAD binding site of LpdA in **(A)** wild type, and **(B)** mutated structure. The protein structure was colored in green, Flavin adenine dinucleotide (FAD) and amino acid residue at codon 44 was shown in ball-and-stick format. Targeted amino acid residues at codon 44 were labeled in three-letter code.

The effects of *lpdA* V44I and 6-bp inframe deletion at *cobM* were assessed by *in silico* simulations. Missense mutation at *lpdA* V44I was identified in the post-treatment strain. Multiple sequence alignment result showed that Val44 was highly conserved across all orthologs selected from MTBC and NTM species in this study (Supplementary Figure 1). X-ray crystallography structure of LpdA (1XDI) in complex with flavin adenine dinucleotide (FAD) was downloaded from RCSB-PDB (Argyrou et al., 2004) and passed to DUET for structural analysis. The protein structure of LpdA showed that Val44 was located next to the FAD binding site (Figure 3). The negative free energy change (ΔΔG = −1.005 Kcal/mol) calculated by DUET has further suggested protein destabilization caused by V44I missense mutation.

A 6-bp inframe deletion in CobM at codon 69–70 was identified in post-treatment strain, which removed Ala69-Asp70 from the resultant protein sequence. Multiple sequence alignment revealed that while Ala69 was highly conserved across different mycobacterial species, the residue at codon 70 could vary from aspartate to histadine in *Mycobacterium avium complex* (MAC) and *Mycobacterium marinum* strains (Supplementary Figure 1). In addition, [Ala-Asp] unit was repeated for three times at codon 67–72, and such repeat was unique to MTBC species. Due to the nature of the indel mutation and the absence of available X-ray crystal structure, sequence-based analysis using PROVEAN was employed to predict the functional effect of the 6-bp inframe deletion. A PROVEAN score of −9.429 was obtained, and hence the deletion was regarded as deleterious to the resultant CobM function.

## Discussion

This is the first study describing the stepwise evolution of drug resistance and growth fitness in MDR-TB during *in vivo* challenge of anti-TB drugs. In this study, we focused on a pair of MDR-TB clinical isolates obtained from the same patient within a 4 months interval of anti-TB therapy. The post-treatment strain exhibited improved *in vitro* growth rate and elevated resistance toward INH, RFB, and EMB when compared to the pre-treatment strain. The clonal relationship between the two MDR-TB clinical isolates was confirmed by identical MIRU-VNTR and spoligotyping patterns, which indicated that the post-treatment strain was developed from pre-treatment strain during the course of anti-TB treatment. Since these two strains were clonally related, comparative genomic analysis could potentially filter out all the lineage-specific mutations, and genetic alterations responsible for the phenotypic differences could be revealed.

The application of SMRT Sequencing in microbial genome assembly has been evaluated extensively in recent years, and it is considered as a robust sequencing platform for MTB genomes with high GC content (Koren et al., 2013; Miyoshi-Akiyama et al., 2015; Rodríguez et al., 2015; Valafar et al., 2015). Despite its high single-pass error rate (15.4–18.7%), previous studies have proved the high consensus accuracy of SMRT Sequencing (Quality Score ≥ Q50) given a sufficient coverage depth (>20X) and the use of error-correction step prior to assembly (Koren et al., 2012). In this study, SMRT Sequencing on both MDR-TB clinical isolates achieved fully closed *de novo* assembled genomes represented by one single contig with uniform coverage. Since the MDR-TB strains were clonally related, it was expected that there would only be a limited number of genetic variations across the two strains. Consistent with our hypothesis, comparative genomic analysis between the two strains only identified 15 polymorphisms throughout the entire bacterial genomes. The result has further demonstrated the consensus accuracy of SMRT Sequencing platform in analyzing the genomic sequences of MTB.

To explain the antibiotic resistance patterns in pre-treatment strain and post-treatment strain, DNA sequences of resistance-related genes were examined from the assembled genomes and the identified mutations were confirmed by Sanger Sequencing. The genes were chosen based on their correlations with antibiotic resistance. Both strains showed concordant genotypic and phenotypic susceptibility profile toward RIF, PZA, STR, OFX, MOX, KAN, AMI, ETH, CYS, and PAS. Both MDR-TB strains were resistant to PZA at 100 mg/L while carrying *pncA* missense mutation G162D. The identical resistance profile of RIF (MIC > 16 mg/L), STR (MIC = 32 mg/L), KAN (MIC > 40 mg/L), and AMI (MIC = 4 mg/L) in both strains could be explained by the missense mutation at *rpoB* S531L, *rpsL* R43K, and *rrs* A1401G respectively (Tracevska et al., 2004; Chan et al., 2007; Jugheli et al., 2009; Jamieson et al., 2014). Meanwhile, the wild-type *folC, gyrA, gyrB*, and *ethA* sequences of both MDR-TB clinical isolates were concordant to the phenotypic susceptibility toward PAS, OFX, and MOX and ETH (Zheng et al., 2013; Zhang and Yew, 2015).

Interestingly, while both strains carried identical *rpoB* S531L missense mutation, the RFB MIC of the post-treatment strain was increased by 16-folds (MIC = 16 mg/L) without any additional mutations at the entire *rpoB* sequence. Promoter mutations at *mabA-inhA* and missense mutations at *katG* codon S315 were known to be the major cause for low level (MIC = 0.2–0.8 mg/L) and high level (MIC > 1 mg/L) INH resistance, respectively (Abe et al., 2008; Ando et al., 2010). Although both MDR-TB clinical isolates only carried *inhA* C-15T mutation, the post-treatment strain demonstrated high level of INH resistance (MIC = 4 mg/L) in the absence of *katG* missense mutations. Similarly, both strains carried *embB* M306V missense mutation in the absence of any missense *ubiA* mutations. The *embB* M306V is predominantly the cause for moderate EMB resistance (MIC = 6–14 mg/L) (Safi et al., 2008; Starks et al., 2009), and can be elevated by 2-folds in the presence of *ubiA* missense mutations (MIC = 16–32 mg/L) (Safi et al., 2013; He et al., 2015). While the pre-treatment strain demonstrated a susceptible to borderline resistance profile toward EMB (MIC = 8 mg/L), the post-treatment strain was resistant to high level EMB resistance with 4-fold elevation in EMB MIC (MIC = 32 mg/L). The discrepancies in the genotypic and phenotypic resistance patterns have hinted the possibility of novel antibiotic resistance development pathways. It is also notable that the post-treatment strain demonstrated an improved *in vitro* growth rate when compared to the pre-treatment strain, which was contrary to general belief that drug resistance development in bacteria is often associated with a fitness deficit. Multiple studies have demonstrated that bacterial fitness could be potentially restored by the presence of compensatory mutations (Comas et al., 2011; He et al., 2015). Our result has further suggested the acquisition of compensatory mutations in the post-treatment strain that restored its growth fitness during the development of additional antibiotic resistance.

Further comparative genomic analysis identified three additional polymorphisms between the two MDR-TB clinical isolates. To investigate the importance of these polymorphisms, multiple protein sequence alignment was performed using orthologs from diverse pathogenic mycobacteria strains representing different lineages of their respective species, which covered most of the pathogenic mycobacterial species commonly identified in Hong Kong. We hypothesized that residue essential for protein functions should be residing in highly conserved regions across different species. For residues that were non-essential to the protein function, polymorphisms would occur even among different lineages of the same mycobacterial species. In other words, if the polymorphisms identified in SMRT Sequencing were located within conserved regions, the mutation is very likely to have significant impact on the resultant protein function.

The first polymorphism was a single nucleotide deletion at codon 120 (nucleotide position 360) in *rv0888* for pre-treatment strain, resulting in the truncation of Rv0888 from 490 residues to 136 residues. Rv0888 is an extracellular nuclease highly active in degrading various types of nucleic acids. The activity of extracellular nuclease could be essential for bacterial virulence, utilization of extracellular DNA as energy source and the degradation of neutrophil DNA extracellular traps (Dang et al., 2016). Moreover, Rv0888 could utilize sphingomyelin from host cell as an additional carbon and energy source during intracellular replication (Speer et al., 2015). Site-directed mutagenesis conducted by Dang et al. demonstrated that His353, Asp387, and Asp438 were critical to Rv0888 nuclease activity (Dang et al., 2016). Truncation of Rv0888 causes the deletion of the aforementioned functional amino acids, thus renders the protein non-functional in pre-treatment strain. The restoration of frameshift mutation back to a functional protein in post-treatment strain could potentially indicate the importance of Rv0888 during the stepwise development of MDR-TB.

The second polymorphism was *lpdA* V44I missense mutation in post-treatment strain. Using multiple sequence alignment result, we demonstrated that V44I was occurring at a highly conserved region in LpdA. Further structural-based analysis demonstrated that V44I was a mutation near the FAD binding site of LpdA and was able to cause protein destabilization. The *lpdA* gene encodes for probable lipoamide dehydrogenase (Argyrou and Blanchard, 2001; Argyrou et al., 2004). Previous reports showed that LpdA belongs to oxidoreductase family which utilizes NADP/NADPH^+^ to maintain the pool of reduced pyridine nucleotides in MTB. LpdA was also suggested to be a quinone reductase that transfers reducing equivalents from the reduced pyridine nucleotide pool to the electron transport chain and protects the bacteria from oxidative stress in *in vivo* conditions (Akhtar et al., 2006; Zheng et al., 2008). The effect of LpdA to drug resistance development is currently unknown. The involvement of LpdA in maintaining the redox equilibrium of MTB might suggest a potential association of LpdA with drug that requires bacterial catalase activation such as INH. Further experimental validations would be required to identify the association of *lpdA* V44I mutation with the elevated INH resistance in post-treatment strain.

The third polymorphism was a deleterious *cobM* inframe deletion at codon 69–70 in post-treatment strain. The exact functional role of CobM is still currently unknown, but it is believed that the gene encodes for Precorrin-4 C (11)-methyltransferase which catalyze the methylation of C-11 in precorrin-4 to precorrin-5 during Vitamin B12 metabolism in MTB (Young et al., 2015), meaning that the gene function could be associated with the bacterial growth rate. Interestingly, the 6-bp inframe deletion occurred at *cobM* codon 69–70 would removed one of the three [Ala-Asp] repeating units at codon 67–72 in post-treatment strain. The occurrence of such inframe deletion specifically in post-treatment strain strongly indicated the potential correlation between the mutation and the improvement of the survival fitness for the post-treatment strain under antibiotic selective pressure.

In this study, 11 polymorphisms identified by SMRT Sequencing were located within a 100 bp repetitive region at the center of PE-PGRS2. Due to the high GC content and the repetitive nature of the gene, we were unable to perform confirmatory Sanger sequencing to verify the mutations in PE-PGRS2. Nevertheless, the inability of confirmatory Sanger Sequencing at PE-PGRS2 in this study has further highlighted the effectiveness of SMRT Sequencing in analyzing the whole genome of MTB. Recent studies have illustrated the potential importance of PE-PGRS and PE-PPE in related to MTB pathogenicity (Kohli et al., 2012; Fishbein et al., 2015). However, the gene family of PE-PGRS were known to be highly variable with a unusually high GC content, they are prone to high frequency of sequencing errors and the effect of the mutations were often difficult to be predicted (Mcevoy et al., 2012; Golby et al., 2013; Tyler et al., 2016). When analyzing the whole genome of MTB using short read sequencing platform, gaps were often generated at regions with repetitive elements, in particularly within PE-PGRS, PE-PPE, *cysA* gene, and insertion elements (Gurjav et al., 2016). Unlike short read sequencing, reads generated by SMRT Sequencing were ~10–20 kbp, which can effectively covers the repetitive regions in MTB genome. The long sequencing read of SMRT Sequencing has greatly simplify the procedures for identifying mutations at PE-PGRS and PE-PPE, and thus genetic makeup of MTB could be investigated in greater details.

There are limitations in this study. Only one pair of MDR-TB clinical isolates was included for comparative analysis. The absence of additional comparison groups was due to the fact that stepwise development of MDR-TB clinical isolates with elevated drug resistance profile is extremely rare in regions adopting Direct Observed Treatment, Short Course regimen. Although stepwise elevation of drug resistance can also be achieved by inductive mutagenesis and clonal selection, these strains were derived in laboratory and might not reflect the actual situation in clinical settings (Safi et al., 2013; Smith et al., 2013). On the other hand, even when strains of the same lineage were used for comparison, it would still result in thousands of potential mutations and further extensive experimental validations would be required. The clonal relatedness of pre-treatment and post-treatment strain means that lineage-specific mutations could be extensively removed during comparative genomic analysis, and the mutations responsible for the phenotypic differences could be revealed.

In this study, growth fitness of the two MDR-TB strains were evaluated by *in vitro* growth curve experiment. Previous studies measured the relative fitness of TB strains using pairwise growth assays, in which the *in vitro* growth of bacteria were analyzed by automated liquid culture systems such as MGIT 960 System and MB/BacT ALERT 3D System (O'sullivan et al., 2010; Safi et al., 2013; Satta et al., 2016). In this study the growth rate of MDR-TB strains were continuously monitored every 48–72 h over a period of 14 days. By using the *in vitro* growth curve, we were able to provide a much more comprehensive evaluation on the growth fitness of MDR-TB at different time-point.

This study has demonstrated that the phenotypic resistance profiles of MDR-TB clinical isolates could be explained by the well-characterized genetic mutations at resistance-related genes. However, further experimental validations are still required to delineate the causal relationship between the newly identified mutations and MTB physiology. In addition, although we have demonstrated the different *in vitro* growth rate of the two MDR-TB clinical isolates, macrophage challenge experiments would be done to fully evaluate their *in vivo* growth fitness. The mutated genes would be transformed into both pre-treatment and post-treatment strains by electroporation. The antibiotic susceptibility profiles and the growth fitness of the transformed strains will then be examined in detail to prove the role of the mutations for the stepwise development of MDR-TB.

In conclusion, our results described the robustness of SMRT Sequencing in identifying mutations among MDR-TB clinical isolates. Comparative genomic analysis between the two clonally related MDR-TB revealed additional mutations at *lpdA, cobM*, and *rv0888* that were developed as a result of anti-TB treatment. The underlying genetic alterations involved could explain the improved *in-vitro* growth fitness and the elevated drug resistance of the post-treatment strain when compared to the pre-treatment strain. Our work has suggested novel gene targets that could potentially play an important role in the physiological characteristics among MDR-TB strains.

## Author contributions

KS-SL, GK-HS, KK-GT, SW-CT, and RR were responsible for planning and conducting the experimental works. P-LH, SS-YW provided the clinical support to this study. WZ and OC-KM provided technical support to PacBio Sequencing. W-CY is the corresponding author and supervise the project.

### Conflict of interest statement

The authors declare that the research was conducted in the absence of any commercial or financial relationships that could be construed as a potential conflict of interest.
