# Supplementary material for: Comparative Genomic Analysis of Two Clonally Related Multidrug Resistant Mycobacterium tuberculosis by Single Molecule Real Time Sequencing
Source: Front Cell Infect Microbiol. 2017 Nov 15;7:478. doi: 10.3389/fcimb.2017.00478 (PMC5694780; doi:10.3389/fcimb.2017.00478)
Supplement: Supplementary file 1 [file Image1.PDF]

Supplementary Figure 1. Multiple protein sequence alignment result of *LpdA, CobM* and Rv0888. Multiple sequence alignment for the first 100 residues for (A) LpdA*;* (B) CobM and (C) Rv0888. Percentage identities at each residue were shown by the intensity of blue color. Targeted residues were highlighted in Red (conserved residues) and in Orange (unconserved residues). Ortholog sequences were searched and obtained from *Mycobacterium tuberculosis* (n=21); *Mycobacterium bovis/BCG* (n=7); *Mycobacterium canettii* (n=5); *Mycobacterium microti* (n=1); *Mycobacterium africanum* (n-1); *Mycobacterium avium* (n=8); *Mycobacterium intracellulare* (n=3); *Mycobacterium paraintracellulare (*n=1); *Mycobacterium yongonense* (n=1); *Mycobacterium indicus pranii* (n=1); *Mycobacterium marinum* (n=1); *Mycobacterium kansasii* (n=3); *Mycobacterium abscessus* (n=10). For LpdA, the genes demonstrated highly conserved region at codon 44. Whereas for CobM, the Ala69 was highly conserved across different mycobacterial species, the residue at codon 70 could vary from aspartate to histadine in *Mycobacterium avium complex* (MAC) and *Mycobacterium marinum* strains. Moreover, [Ala-Asp] unit was repeated for three times at codon 67-72, and such repeat was unique to *Mycobacterium tuberculosis complex species*. For Rv0888, multiple sequence alignment showed that the first 100 residues were only conserved among *Mycobacterium tuberculosis* complex species, while the rest of the protein would be truncated with the frameshift mutation at codon 120 identified in the post-treatment strain.

A.

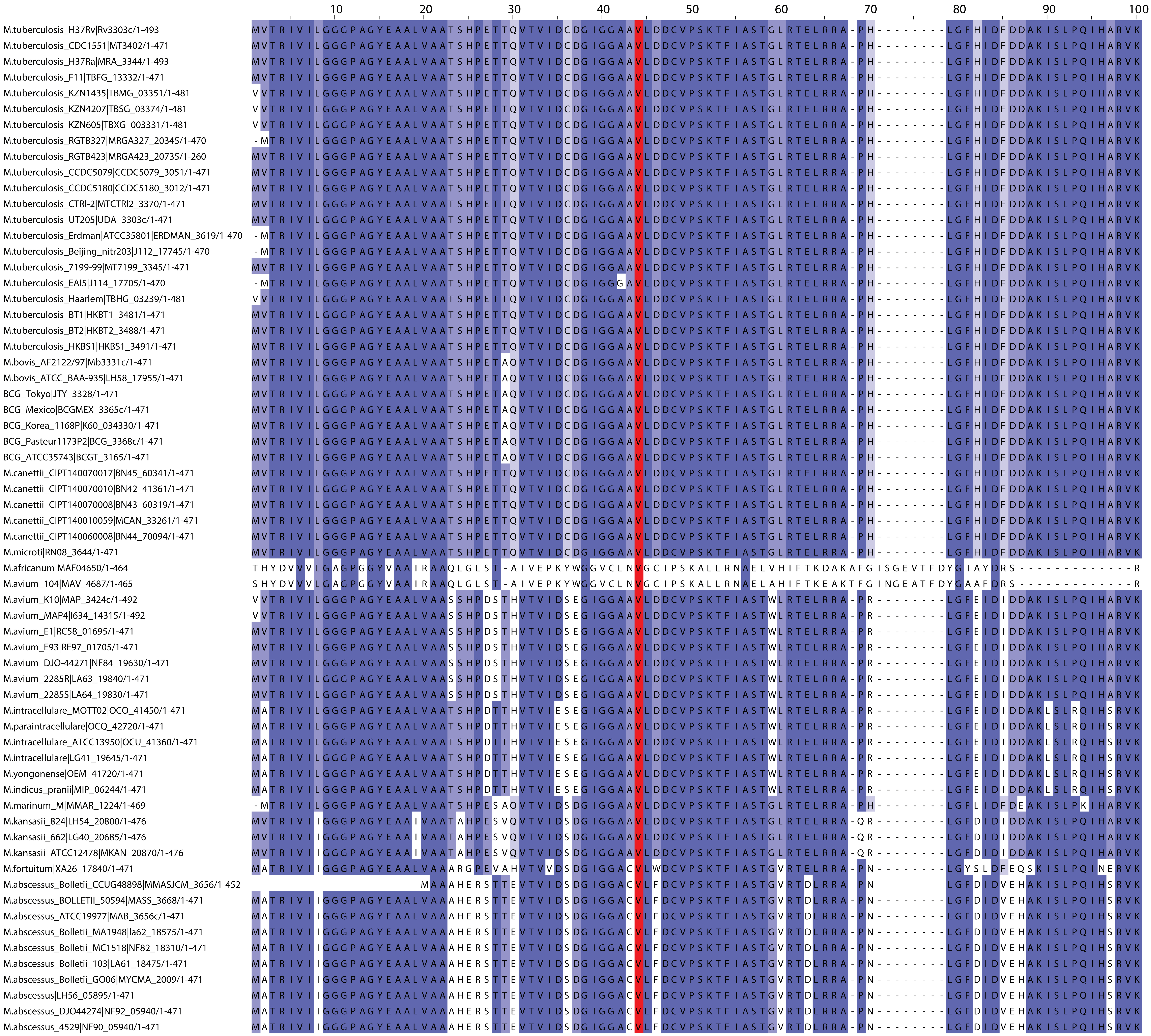

10
20
30
40
50
60
70
80
90
100
M.tuberculosis_H37Rv|Rv3303c/1-493
M.tuberculosis_CDC1551|MT3402/1-471
M.tuberculosis_H37Ra|MRA_3344/1-493
M.tuberculosis_F11|TBFG_13332/1-471
M.tuberculosis_KZN1435|TBMG_03351/1-481
M.tuberculosis_KZN4207|TBSG_03374/1-481
M.tuberculosis_KZN605|TBXG_003331/1-481
M.tuberculosis_RGTB327|MRGA327_20345/1-470
M.tuberculosis_RGTB423|MRGA423_20735/1-260
M.tuberculosis_CCDC5079|CCDC5079_3051/1-471
M.tuberculosis_CCDC5180|CCDC5180_3012/1-471
M.tuberculosis_CTRI-2|MTCTRI2_3370/1-471
M.tuberculosis_UT205|UDA_3303c/1-471
M.tuberculosis_Erdman|ATCC35801|ERDMAN_3619/1-470
M.tuberculosis_Beijing_nitr203|J112_17745/1-470
M.tuberculosis_7199-99|MT7199_3345/1-471
M.tuberculosis_EAI5|J114_17705/1-470
M.tuberculosis_Haarlem|TBHG_03239/1-481
M.tuberculosis_BT1|HKBT1_3481/1-471
M.tuberculosis_BT2|HKBT2_3488/1-471
M.tuberculosis_HKBS1|HKBS1_3491/1-471
M.bovis_AF2122/97|Mb3331c/1-471
M.bovis_ATCC_BAA-935|LH58_17955/1-471
BCG_Tokyo|JTY_3328/1-471
BCG_Mexico|BCGMEX_3365c/1-471
BCG_Korea_1168P|K60_034330/1-471
BCG_Pasteur1173P2|BCG_3368c/1-471
BCG_ATCC35743|BCGT_3165/1-471
M.canettii_CIPT140070017|BN45_60341/1-471
M.canettii_CIPT140070010|BN42_41361/1-471
M.canettii_CIPT140070008|BN43_60319/1-471
M.canettii_CIPT140010059|MCAN_33261/1-471
M.canettii_CIPT140060008|BN44_70094/1-471
M.microti|RN08_3644/1-471
M.africanum|MAF04650/1-464
M.avium_104|MAV_4687/1-465
M.avium_K10|MAP_3424c/1-492
M.avium_MAP4|I634_14315/1-492
M.avium_E1|RC58_01695/1-471
M.avium_E93|RE97_01705/1-471
M.avium_DJO-44271|NF84_19630/1-471
M.avium_2285R|LA63_19840/1-471
M.avium_2285S|LA64_19830/1-471
M.intracellulare_MOTT02|OCO_41450/1-471
M.paraintracellulare|OCQ_42720/1-471
M.intracellulare_ATCC13950|OCU_41360/1-471
M.intracellulare|LG41_19645/1-471
M.yongonense|OEM_41720/1-471
M.indicus_pranii|MIP_06244/1-471
M.marinum_M|MMAR_1224/1-469
M.kansasii_824|LH54_20800/1-476
M.kansasii_662|LG40_20685/1-476
M.kansasii_ATCC12478|MKAN_20870/1-476
M.fortuitum|XA26_17840/1-471
M.abscessus_Bolletii_CCUG48898|MMASJCM_3656/1-452
M.abscessus_BOLLETII_50594|MASS_3668/1-471
M.abscessus_ATCC19977|MAB_3656c/1-471
M.abscessus_Bolletii_MA1948|la62_18575/1-471
M.abscessus_Bolletii_MC1518|NF82_18310/1-471
M.abscessus_Bolletii_103|LA61_18475/1-471
M.abscessus_Bolletii_GO06|MYCMA_2009/1-471
M.abscessus|LH56_05895/1-471
M.abscessus_DJO44274|NF92_05940/1-471
M.abscessus_4529|NF90_05940/1-471

B.

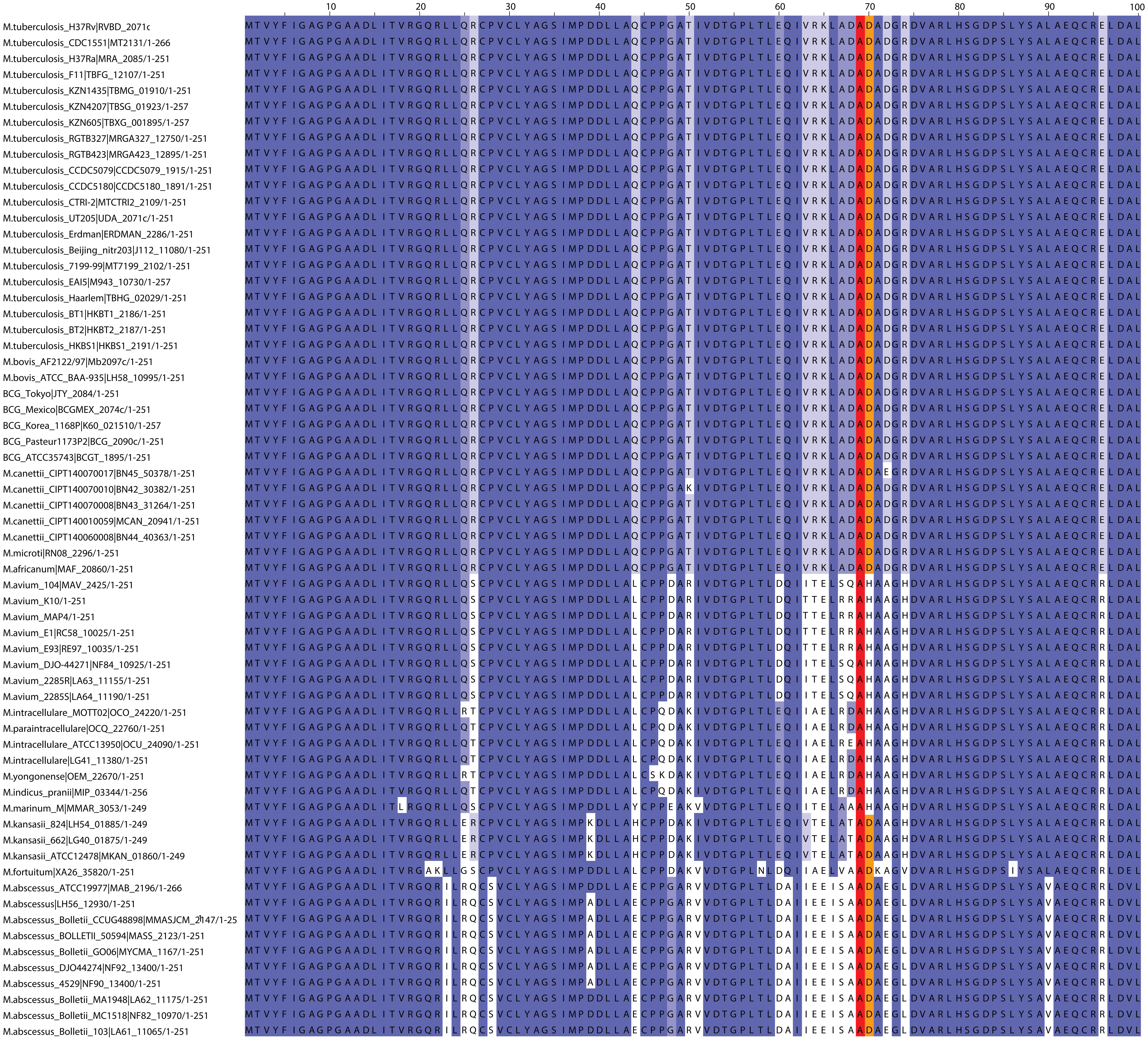

10
20
30
40
50
60
70
80
90
100
M.tuberculosis_H37Rv|RVBD_2071c
M.tuberculosis_CDC1551|MT2131/1-266
M.tuberculosis_H37Ra|MRA_2085/1-251
M.tuberculosis_F11|TBFG_12107/1-251
M.tuberculosis_KZN1435|TBMG_01910/1-251
M.tuberculosis_KZN4207|TBSG_01923/1-257
M.tuberculosis_KZN605|TBXG_001895/1-257
M.tuberculosis_RGTB327|MRGA327_12750/1-251
M.tuberculosis_RGTB423|MRGA423_12895/1-251
M.tuberculosis_CCDC5079|CCDC5079_1915/1-251
M.tuberculosis_CCDC5180|CCDC5180_1891/1-251
M.tuberculosis_CTRI-2|MTCTRI2_2109/1-251
M.tuberculosis_UT205|UDA_2071c/1-251
M.tuberculosis_Erdman|ERDMAN_2286/1-251
M.tuberculosis_Beijing_nitr203|J112_11080/1-251
M.tuberculosis_7199-99|MT7199_2102/1-251
M.tuberculosis_EAI5|M943_10730/1-257
M.tuberculosis_Haarlem|TBHG_02029/1-251
M.tuberculosis_BT1|HKBT1_2186/1-251
M.tuberculosis_BT2|HKBT2_2187/1-251
M.tuberculosis_HKBS1|HKBS1_2191/1-251
M.bovis_AF2122/97|Mb2097c/1-251
M.bovis_ATCC_BAA-935|LH58_10995/1-251
BCG_Tokyo|JTY_2084/1-251
BCG_Mexico|BCGMEX_2074c/1-251
BCG_Korea_1168P|K60_021510/1-257
BCG_Pasteur1173P2|BCG_2090c/1-251
BCG_ATCC35743|BCGT_1895/1-251
M.canettii_CIPT140070017|BN45_50378/1-251
M.canettii_CIPT140070010|BN42_30382/1-251
M.canettii_CIPT140070008|BN43_31264/1-251
M.canettii_CIPT140010059|MCAN_20941/1-251
M.canettii_CIPT140060008|BN44_40363/1-251
M.microti|RN08_2296/1-251
M.africanum|MAF_20860/1-251
M.avium_104|MAV_2425/1-251
M.avium_K10/1-251
M.avium_MAP4/1-251
M.avium_E1|RC58_10025/1-251
M.avium_E93|RE97_10035/1-251
M.avium_DJO-44271|NF84_10925/1-251
M.avium_2285R|LA63_11155/1-251
M.avium_2285S|LA64_11190/1-251
M.intracellulare_MOTT02|OCO_24220/1-251
M.paraintracellulare|OCQ_22760/1-251
M.intracellulare_ATCC13950|OCU_24090/1-251
M.intracellulare|LG41_11380/1-251
M.yongonense|OEM_22670/1-251
M.indicus_pranii|MIP_03344/1-256
M.marinum_M|MMAR_3053/1-249
M.kansasii_824|LH54_01885/1-249
M.kansasii_662|LG40_01875/1-249
M.kansasii_ATCC12478|MKAN_01860/1-249
M.fortuitum|XA26_35820/1-251
M.abscessus_ATCC19977|MAB_2196/1-266
M.abscessus|LH56_12930/1-251
M.abscessus_Bolletii_CCUG48898|MMASJCM_2147/1-25
M.abscessus_BOLLETII_50594|MASS_2123/1-251
M.abscessus_Bolletii_GO06|MYCMA_1167/1-251
M.abscessus_DJO44274|NF92_13400/1-251
M.abscessus_4529|NF90_13400/1-251
M.abscessus_Bolletii_MA1948|LA62_11175/1-251
M.abscessus_Bolletii_MC1518|NF82_10970/1-251
M.abscessus_Bolletii_103|LA61_11065/1-251

C.

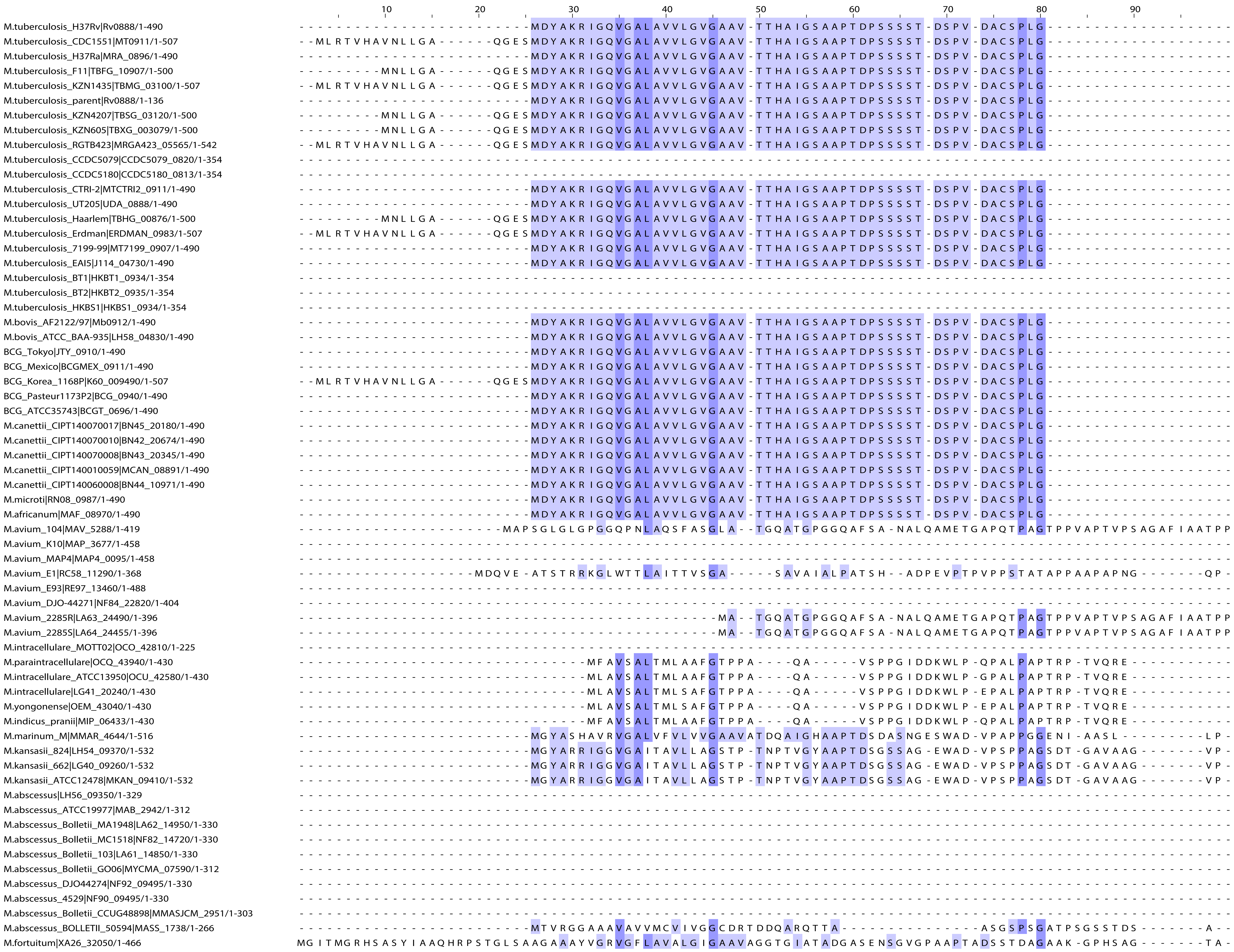
10
20
30
40
50
60
70
80
90
M.tuberculosis_H37Rv|Rv0888/1-490
M.tuberculosis_CDC1551|MT0911/1-507
M.tuberculosis_H37Ra|MRA_0896/1-490
M.tuberculosis_F11|TBFG_10907/1-500
M.tuberculosis_KZN1435|TBMG_03100/1-507
M.tuberculosis_parent|Rv0888/1-136
M.tuberculosis_KZN4207|TBSG_03120/1-500
M.tuberculosis_KZN605|TBXG_003079/1-500
M.tuberculosis_RGTB423|MRGA423_05565/1-542
M.tuberculosis_CCDC5079|CCDC5079_0820/1-354
M.tuberculosis_CCDC5180|CCDC5180_0813/1-354
M.tuberculosis_CTRI-2|MTCTRI2_0911/1-490
M.tuberculosis_UT205|UDA_0888/1-490
M.tuberculosis_Haarlem|TBHG_00876/1-500
M.tuberculosis_Erdman|ERDMAN_0983/1-507
M.tuberculosis_7199-99|MT7199_0907/1-490
M.tuberculosis_EAI5|J114_04730/1-490
M.tuberculosis_BT1|HKBT1_0934/1-354
M.tuberculosis_BT2|HKBT2_0935/1-354
M.tuberculosis_HKBS1|HKBS1_0934/1-354
M.bovis_AF2122/97|Mb0912/1-490
M.bovis_ATCC_BAA-935|LH58_04830/1-490
BCG_Tokyo|JTY_0910/1-490
BCG_Mexico|BCGMEX_0911/1-490
BCG_Korea_1168P|K60_009490/1-507
BCG_Pasteur1173P2|BCG_0940/1-490
BCG_ATCC35743|BCGT_0696/1-490
M.canettii_CIPT140070017|BN45_20180/1-490
M.canettii_CIPT140070010|BN42_20674/1-490
M.canettii_CIPT140070008|BN43_20345/1-490
M.canettii_CIPT140010059|MCAN_08891/1-490
M.canettii_CIPT140060008|BN44_10971/1-490
M.microti|RN08_0987/1-490
M.africanum|MAF_08970/1-490
M.avium_104|MAV_5288/1-419
M.avium_K10|MAP_3677/1-458
M.avium_MAP4|MAP4_0095/1-458
M.avium_E1|RC58_11290/1-368
M.avium_E93|RE97_13460/1-488
M.avium_DJO-44271|NF84_22820/1-404
M.avium_2285R|LA63_24490/1-396
M.avium_2285S|LA64_24455/1-396
M.intracellulare_MOTT02|OCO_42810/1-225
M.paraintracellulare|OCQ_43940/1-430
M.intracellulare_ATCC13950|OCU_42580/1-430
M.intracellulare|LG41_20240/1-430
M.yongonense|OEM_43040/1-430
M.indicus_pranii|MIP_06433/1-430
M.marinum_M|MMAR_4644/1-516
M.kansasii_824|LH54_09370/1-532
M.kansasii_662|LG40_09260/1-532
M.kansasii_ATCC12478|MKAN_09410/1-532
M.abscessus|LH56_09350/1-329
M.abscessus_ATCC19977|MAB_2942/1-312
M.abscessus_Bolletii_MA1948|LA62_14950/1-330
M.abscessus_Bolletii_MC1518|NF82_14720/1-330
M.abscessus_Bolletii_103|LA61_14850/1-330
M.abscessus_Bolletii_GO06|MYCMA_07590/1-312
M.abscessus_DJO44274|NF92_09495/1-330
M.abscessus_4529|NF90_09495/1-330
M.abscessus_Bolletii_CCUG48898|MMASJCM_2951/1-303
M.abscessus_BOLLETII_50594|MASS_1738/1-266
M.fortuitum|XA26_32050/1-466
